# Immune Responses in Pigs Induced by Recombinant DNA Vaccine Co-Expressing Swine IL-18 and Membrane Protein of Porcine Reproductive and Respiratory Syndrome Virus

**DOI:** 10.3390/ijms13055715

**Published:** 2012-05-11

**Authors:** Xiaodong Zhang, Xiaoli Wang, Lianzhi Mu, Zhuang Ding

**Affiliations:** College of Animal Science and Veterinary Medicine, and Key Laboratory of Zoonosis Research, Ministry of Education, Institute of Zoonosis, Jilin University, Changchun 130062, China; E-Mails: zhang_xd@jlu.edu.cn (X.Z.); jackcaas@163.com (X.W.); dawnezh@163.com (L.M.)

**Keywords:** PRRS, M protein, IL-18, immune response, DNA vaccine

## Abstract

In this study, two DNA vaccines, which express the membrane (M) protein of porcine respiratory and reproductive syndrome virus (PRRSV) (pEGFP-M) and co-express both M and swine IL-18 (pEGFP-IL18-M), were constructed and their abilities to induce humoral and cellular responses in piglets were comparatively evaluated. Experimental results showed that both recombinant DNA vaccines could not elicit neutralizing antibodies in the immunized piglets. However, both DNA vaccines elicited Th1-biased cellular immune responses. Notably, pigs immunized with the plasmid pEGFP-IL18-M developed significantly higher levels of IFN-γ and IL-2 production response and stronger specific T-lymphocyte proliferation response than the pigs inoculated with the plasmids pEGFP-M and pEGFP-IL18 (*P* < 0.05). These results illustrated that co-expression of M and IL-18 proteins could significantly improve the potency of DNA vaccination on the activation of vaccine-induced virus-specific cell-mediated immune responses in pigs, which may be used as a strategy to develop a new generation of vaccines against highly pathogenic PRRSV.

## 1. Introduction

Porcine reproductive and respiratory syndrome (PRRS) is the most economically significant viral disease in the swine industry. It is characterized by respiratory illness in all age groups of swine leading to death in some younger pigs and severe reproductive problems in breeding age females. China not only is the biggest country of pig and pork production but also is the largest consumption market of pork in the world. Since PRRS outbreaks were first recognized in an intensive pig farms in North China at the end of 1995 [1], the disease has brought considerable loss to China’s swine industry each year although no exact amount has been evaluated. Since then, the North American type PRRSV has been predominantly circulating in China. In particular, a highly pathogenic PRRS broke out in the central region of China and has quickly spread throughout the country since 2006 [2,3]. The newly appearing PRRS caused high morbidity of 50–100% and a mortality rate of 20–100%. The high mortality in grown pigs is unusual and led to a countrywide epidemic of atypical PRRS. The highly virulent atypical PRRS occurred in many herds vaccinated with commercial PRRS vaccines, suggesting that the commercial vaccines were unable to protect pigs from PRRS [4].

The causative agent, PRRS virus (PRRSV), is a positive sense RNA virus that groups within the order Nidovirales, family Arteriviridae. The genome of PRRSV is approximately 15 kb in length and contains nine open reading frames (ORFs). Currently, PRRSV includes two major antigenic types, the European type and the North American type. ORF1a and ORF1b encode viral replicase polyproteins that are immediately translated upon viral entry, and proteolytically processed by virally encoded proteinases into at least 13 mature nonstructural proteins (Nsps) [5–7]. ORF2a, ORF2b, ORF3 and ORF4 code for structural proteins GP2a, GP2b (E), GP3, GP4, respectively [8]. ORFs 5–7 encode three major structural proteins of virion: the envelope glycoprotein GP5, the non-glycosylated membrane protein (M) and the nucleocapsid protein (N), respectively [9]. Recently, a novel structural protein encoded by ORF5a was confirmed to play a significant role in PRRSV infection [10,11].

The non-glycosylated membrance protein (18–19 Da) encoded by ORF6 contains 1–2 putative glycosylation sites and neutralizing epitopes [9,12]. The M protein is the most conserved structural protein between North American and European isolates. Of all the structural proteins, the M protein is the most potent inducer of T lymphocyte proliferation [13]. Thus, M protein is considered very important in the arousal of cellular immune responses against PRRSV infection and may be an excellent candidate protein in the bioengineering of vaccine [14]. Vaccination with plasmid DNA encoding M protein may provide partial protection in pigs.

In order to accelerate and magnify immune efficiency to PRRSV vaccine, several kinds of cytokines as adjuvants have been utilized to improve immune responses to PRRSV vaccines, including IL-2 [15,16], IL-12 [17], CD40 ligand [18], GM-CSF [19] and IL-18 [20]. Among them, Interleukin-18 (IL-18) has been widely used as adjuvant to enhance immune responses of many vaccine antigens. IL-18, also known as IFN-γ inducing factor, is a pleiotropic cytokine that plays an important role in both innate and acquired immunity [21,22]. But its function is mainly reflected in the T cell and the enhancement of cell-mediated immunity. In particular, IL-18 enhances cytotoxicity of NK cells and activator of the proliferation and the activity of T and NK cells [23,24]. Therefore, it is a good candidate for the modulation of immune responses and is employed as a DNA vaccine adjuvant in this study.

In this study, two DNA vaccine constructs, expressing M protein of PRRSV alone (pEGFP-M) and co-expressing M and swine IL-18 proteins (pEGFP-IL18-M), were constructed and comparatively evaluated for their abilities to induce humoral and cellular responses in pigs, and what’s more, the effect of swine IL-18 in the modulation of a DNA vaccine-induced immune responses could be known.

## 2. Results

### 2.1. Construction and Expression of Recombinant Eukaryotic Plasmids

**Figure 1:**
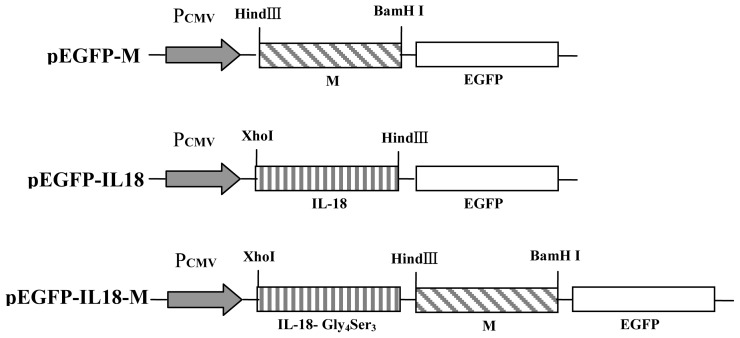
Schematic diagrams of recombinant plasmids—pEGFP-M, pEGFP-IL18 and pEGFP-IL18-M. pEGFP-N1 was used for the construction of the recombinant plasmids.

As shown in Figure 1, under the control of CMV promoter, the recombinant plasmids pEGFP-M and pEGFP-IL18-M containing IL-18 and M genes with 15 amino acids linker (GGGGS)_3_ between them were constructed. The sequence analysis showed that the nucleotide sequences of the inserted genes in recombinant plasmids were the same as the original sequences and they were also inserted in the correct order in the plasmids.

**Figure 2:**
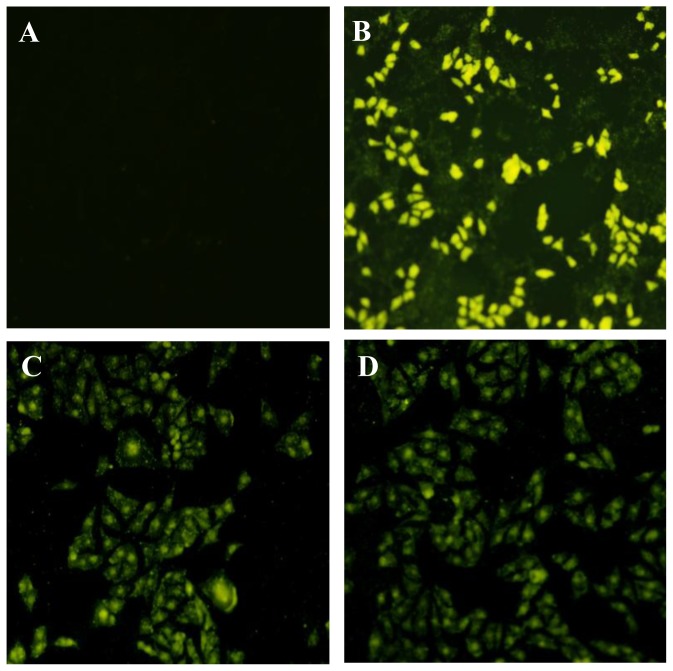
Fluorescent photos of the transfected MARC-145 cells. (**A**) The picture of negative control; (**B**) Green fluorescence images of cells transfected with pEGFP-N1; (**C**) Green fluorescence images of cells transfected with pEGFP-M; and (**D**) Green fluorescence images of cells transfected with pEGFP-IL18-M.

**Figure 3:**
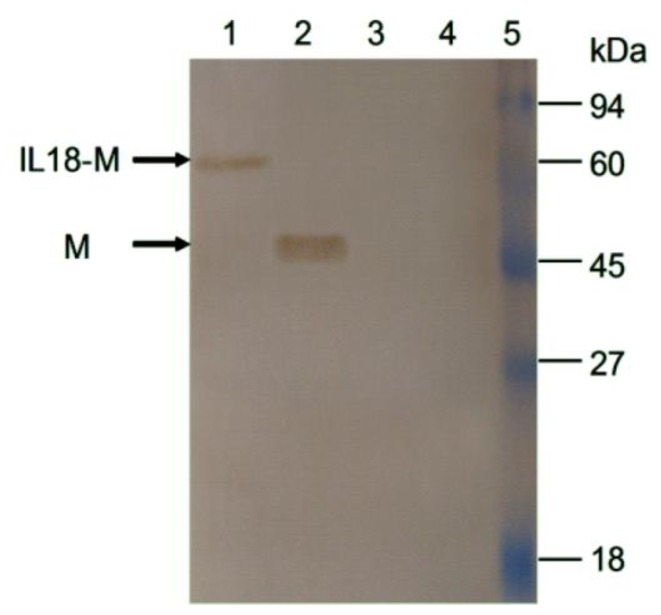
Western blot analysis of lysates of recombinant eukaryon expression plasmids transfected cells, pEGFP-IL18-M (lane 1), pEGFP-M (lane 2), pEGFP-N1 (lane 3), and negative control (lane 4). MARC-145 cells lysates without transfection were used as the control. Protein marker is shown on the right side.

To determine whether the foreign genes could be expressed or not, MARC-145 cells were transfected with recombinant plasmids and the fluorescence were detected, because the cloned genes M and IL18-M were respectively co-expressed as fusions with the *N*-terminus of EGFP respectively, as shown in Figure 1. Moreover, the transfected cells were collected and also verified by Western blotting. As shown in Figure 2, bright green fluorescence could be detected in MARC-145 cells respectively transfected with the two recombinant plasmids pEGFP-M and pEGFP-IL18-M, indicating that foreign genes M and M with IL-18 could be exactly expressed in mammalian cells. As shown in Figure 3, two bands, approximately 49 kDa and 67 kDa, were respectively recognized by PRRSV-specific antibody in lysates of pEGFP-M and pEGFP-IL18-M transfected cells, proving that the foreign genes were indeed expressed in mammalian cells.

### 2.2. Humoral Immune Responses

**Figure 4:**
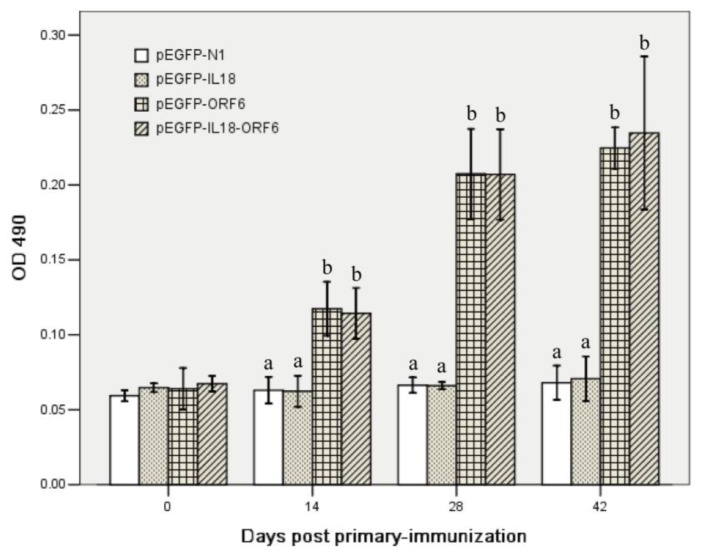
M-specific enzyme-linked immunosorbent assay (ELISA) antibody responses in piglets immunized with different recombinant plasmids. Serum samples (*n* = 5) were collected at various time points and the specific antibodies to porcine respiratory and reproductive syndrome virus (PRRSV) were measured by indirect ELISA. Data are presented as the mean ± S.D. Different letters (a, b) above the columns at the same time point indicate significant difference (*P* < 0.05) between the treatments.

The immunogenicity of recombinant eukaryotic expression plasmids were examined in piglets. As shown in Figure 4, compared with the pigs vaccinated with pEGFP-IL18 and pEGFP-N1, detectable ELISA antibody in pigs vaccinated with pEGFP-M and pEGFP-IL18-M increased significantly, especially after the booster immunization (*P* < 0.05). But no significant difference was observed between the pEGFP-M and pEGFP-IL18-M groups (*P* > 0.05).

To further examine the protective neutralizing antibody, serum neutralizing (SN) antibody titres in the vaccinated pigs were also monitored after primary immunization. Compared with the pigs vaccinated with pEGFP-N1, no specific neutralizing antibodies were detected at all in pigs respectively inoculated with pEGFP-M and pEGFP-IL18-M till 42 days post primary-immunization. Throughout the assay, empty vector vaccination did not display any SN antibody activity.

### 2.3. Lymphocyte Proliferation Responses

**Figure 5:**
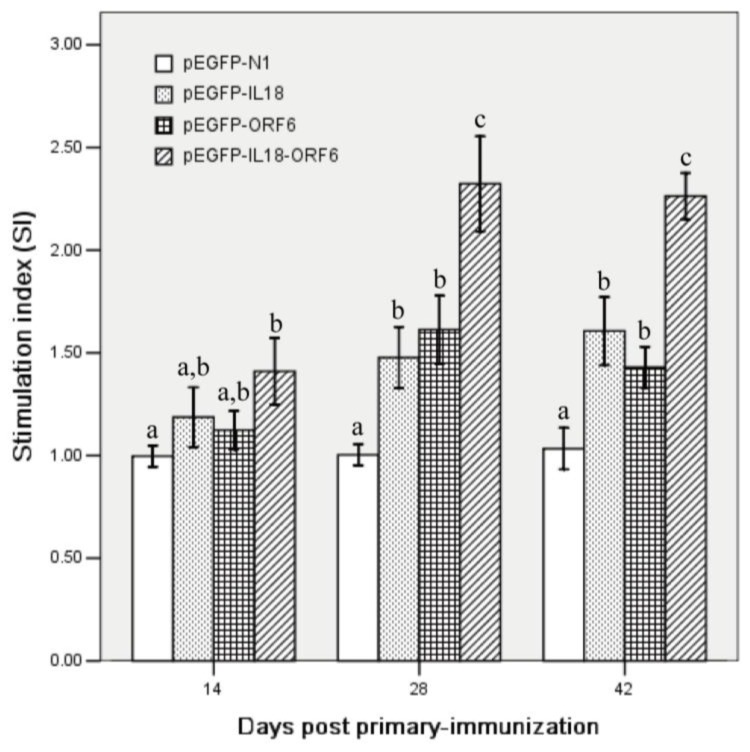
Lymphocyte proliferation responses of piglets immunized with different recombinant plasmids. Blood samples (*n* = 5) were collected at various time-points and were stimulated with PRRSV protein in triplicate. Data are presented as the mean ± S.D. Different letters (a, b, c) above the columns at the same time point indicate significant difference (*P* < 0.05) between the treatments.

The cell-mediated immune response was evaluated through PBMCs proliferation assay performed at 14, 28 and 42 days after primary immunization. As shown in Figure 5, the numerically highest lymphocyte proliferation activity was observed in the group immunized with pEGFP-IL18-M, and after booster immunization, its stimulation index was significantly higher than those of the groups immunized with pEGFP-M and pEGFP-IL18 (*P* < 0.05).

### 2.4. Cellular Immune Response

**Figure 6:**
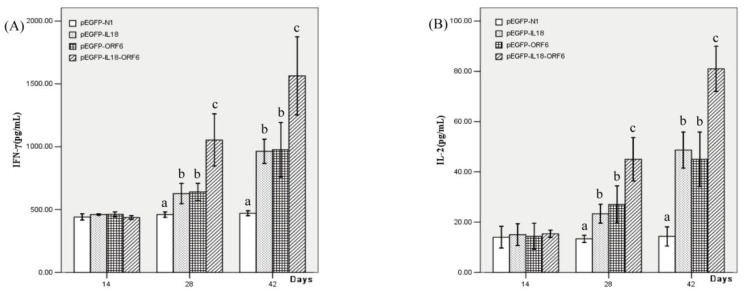
(**A**) IFN-γ level in peripheral blood from piglets inoculated with the recombinant plasmids (**B**) IL-2 level in peripheral blood from piglets inoculated with the recombinant plasmids. Different letters (a, b, c) above the columns at the same time point indicate significant difference (*P* < 0.05) between the treatments.

To further evaluate cellular immune responses, the productions of IFN-γ and IL-2 in peripheral blood from the vaccinated piglets were examined. As shown in Figure 6, after booster immunization the levels of serum IFN-γ and IL-2 were significantly higher in the group that received pEGFP-IL18-M than the levels of serum IFN-γ and IL-2 in any other groups (*P* < 0.05). The results indicated that IL-18 had efficiently enhanced the cellular immune responses to M protein of PRRSV.

## 3. Discussion

Currently no specific treatment is available for PRRS, so vaccination is an efficient strategy to control PRRS. There are two major categories of commercially available PRRSV vaccines: the modified-live virus (MLV) vaccine and killed-virus (KV) vaccine. KV vaccine could only provide partial immune protection to the Chinese highly pathogenic PRRSV [25]. Though PRRSV MLV vaccines confer solid protection against clinical disease induced by homologous infection, they have the potential to revert to virulence [26–28], restricting the application of this vaccination approach. Therefore, improved PRRSV vaccines or new generation vaccines against PRRSV need to be explored. Since IL-18 has previously been demonstrated to have potent adjuvant activity [20], we fused the porcine IL-18 gene with the PRRSV-M gene to enhance the immune responses to the M protein in the immunized pigs, resulting in the recombinant plasmid pEGFP-IL18-M. We not only addressed the adjuvant effect of IL-18 but also focused on the humoral and cellular immune responses generated by the M protein.

Neutralizing antibody is a major component of humoral immunity that plays an important role in protection against PRRSV infection or reinfection, and in prevention or reduction of viral spread from pigs to pigs [29,30]. In this study, experimental results demonstrated that the DNA vaccines pEGFP-IL18-M and pEGFP-M could induce antibodies, but no specific neutralizing antibodies were detected in the sera of vaccinated piglets. Interestingly and coincidently, in previous reports, Kwang *et al*. reported that this absent antibody production was occasionally detected in the pigs immunized with plasmid DNAs expressing the M protein [31], and Zheng *et al*. also reported that no antibodies were detected when experimental mice were only infected with recombinant vaccinia virus expressing M protein alone [14]. Therefore, we concluded that the M protein expressed by plasmid DNA in this case was incompetent to induce protective neutralizing antibody in pigs.

Cell-mediated immunity plays an important role against PRRSV infection. It has become evident that T cell-mediated immunity is essential for effective protection against PRRSV [32,33]. Coincidently, the M protein is the most conserved structural protein and the most potent inducer of T lymphocyte proliferation [13]. And the function of IL-18 (also known as IFN-γ inducing factor) is mainly reflected in the enhancement of cell-mediated immunity and in regulating both Th1- and Th2- driven immune responses [24]. To induce an effective cellular immune response is dependent on the specific cytokines produced during the infection, and the cytokine responses are mainly IFN-γ and to a lesser extent, IL-2 [34]. Furthermore, early studies showed that pigs recovering from experimental PRRSV infection had strong lymphocyte proliferative responses [34,35]. Therefore, in order to evaluate DNA vaccine-induced PRRSV-specific cell-mediated response, we measured T-lymphocyte proliferation response, IFN-γ and IL-2 production responses. The experimental results demonstrated that all recombinant plasmids elicited a cell-mediated immune response, but pigs immunized with pEGFP-IL18-M developed significantly higher level of IFN-γ and IL-2 responses and had significantly stronger specific T-lymphocyte proliferation response than the pigs vaccinated with the pEGFP-M and pEGFP-IL18 (*P* < 0.05). It indicated that compared with the DNA vaccine of expressing M alone, porcine IL-18 when fused with PRRSV M protein had a significantly positive inductive effect on the activation of vaccine-induced virus-specific cellular immune responses in piglets. In addition, the M protein of PRRSV could only serve as a potent antigen for T cell-mediated immune responses but proved insufficient to induce protective neutralizing antibodies by itself.

It has been evident that M protein interacting with GP5 protein—a primary target of neutralizing antibody, can induce both good humoral immunity and cellular immunity. Zheng *et al*. reported that the recombinant vaccinia virus coexpressing the GP5 and M proteins generated more antibodies to the M protein in infected mice [14]. Moreover, Chia *et al.* reported that DNA constructs co-expressing GP5 and M proteins of PRRSV were able to induce a strong PRRSV-specific cell-mediated immunity and antibody mediated immunity responses in pigs [36], and Jiang *et al.* reported that a suicidal DNA vaccine co-expressing a modified GP5 and M proteins of PRRSV also induced protective responses [37]. So co-expression of IL18-M protein and a modified GP5 protein may get better protective immunity than co-expression of M and modified GP5 protein, which would be a good subject for future research. And the identification of T-cell epitopes on the M protein of PRRSV may help us to better understand the cell-mediated immunity in protection against PRRSV.

## 4. Materials and Methods

### 4.1. Virus, Cells and Plasmid

The PRRSV JL/07/SW strain was isolated by our research group from the brains and lungs of field pigs at the acute stage of PRRSV infection in Jilin province China, which was identified as a North American type isolate [38]. After cloning and sequencing its N gene, the result demonstrated that it had 93.8% nucleotide sequence homology with North American PRRSVs, and 53.1% nucleotide sequence homology with European American PRRSV. The virus was propagated and titered on MARC-145 cells which are clones of MA-104 cells highly permissive to PRRSV [39]. The culture method for MARC-145 cells was carried out according to the method of Jiang *et al.* [40]. All expression plasmids were constructed using pEGFP-N1 as the basis vector.

### 4.2. Construction of the Eukaryotic Expression Plasmids

The complete coding sequence of the PRRSV M (ORF6) gene was obtained by RT-PCR from the total RNA extracted from PRRS virus-infected cells, using specific primers (ORF6F: AAGCTTATGGGGTCGTCTCTA and ORF6R: GGATCCGGTTTGGCATATTTAAC). To facilitate the cloning of PCR products into the expression vector (pEGFP-N1), the recognition sequences for Hind III (*AAGCTT*) and BamH I (*GGATCC*) were designed into the corresponding forward and reverse primers. The PCR products were digested with the enzymes and finally inserted into the pEGFP-N1 vector. IL-18 gene was obtained from the total RNA extracted from swine spleen lymphocytes. The extracted RNA was used as template for a RT-PCR reaction with specific primers (IL-18F: GCACTCGAGATGTACTTGGCTA, IL-18R: GCGAAGCTT*GGTGGAGGCGGATCCGGCGGAGGT GGCTCAGGCGGTGGCGGATCT*TTTTGTTTTGA), which contains a flexible linker (Gly_4_Ser)_3_ [41]. The amplification product of IL-18 gene was cloned into the vector pEGFP-N1 by XhoI (*CTCGAG*) and Hind III (*AAGCTT*). For the construction of the eukaryotic expression plasmid pEGFP-IL18-M, the M gene was digested with HindIII and BamH I and subcloned into the plasmid pEGFP-IL18. All recombinant plasmids were confirmed by restriction digestion and sequence analysis.

### 4.3. Identification of Expressions of M and M with IL-18 Proteins through Fluorescent Microscope and Western Blot

MARC-145 cells were cultivated on a concentration of 2.5 × 10^5^ cells/well in a 6-well tissue culture plate. Till the cells reaching approximately 70–80% confluence, the recombinant plasmids pEGFP-IL18-M and pEGFP-M were respectively transfected into MARC-145 cells. Meanwhile the plasmid pEGFP-N1 was transfected into MARC-145 cells, used as positive control. Transfection was performed using LipofectAMINE 2000 Reagent (Invitrogen) according to the manufacturer’s instructions. The transfected cells were washed once with PBS (pH 7.4) at 72 h post transfection to remove non-attached cells and then fluorescences were observed by an inverted fluorescent microscope (Olympus).

For Western bolt analysis, the transfected cells were treated according to the method of Hou *et al.* [42], and finally transferred onto nitrocellulose membrane. Then the membrane was incubated with PRRSV-specific antiserum, followed by horseradish peroxidase-conjugated goat anti-pig IgG, and followed by visualizing with DAB substrate. The untransfected MARC-145 cells lysates were used as negative control.

### 4.4. Immunization of Pigs with the Recombinant Plasmids

Twenty 30-day-old crossbreed (Landrace × local stock) piglets were randomly and averagely separated into four groups and respectively fed in separate rooms. All piglets free of porcine pathogens included as category 1, 2 and 3 animal infectious diseases in Chinese government were also negative for PRRSV tested by enzyme-linked immunosorbent assay (ELISA). Three groups were respectively immunized with 1 mg of each DNA vaccine—plasmids pEGFP-M, pEGFP-IL18 and pEGFP-IL18-M. Another group was immunized with 1 mg of the empty vector pEGFP-N1. Booster immunization was conducted 21 days later. The injection route was direct inoculation of the plasmids into pigs’ neck muscles. Sera samples were collected at 0, 14, 28, 42 days after first immunization for indirect ELISA and serum neutralization assay. Furthermore, at 14, 28 and 42 days after first inoculation, blood samples were collected for lymphocyte proliferation assay and cytokine assay.

### 4.5. Indirect ELISA

Sera collected from experimental piglets at various time points were tested for the detection of specific antibodies by indirect ELISA, using purified SW/07/JL PRRSV as coating antigen. The 96-well ELISA plates were coated overnight at 4 °C with 2 μg/mL of purified virus. Then plates were blocked for 1 h at 37 °C with blocking buffer (2% BSA in PBST) and washed three times with PBST washing buffer. Then sera samples were made a 20-fold dilution in blocking buffer, then added to wells (100 μL per well) in duplicate, and incubated for 1 h at 37 °C. Followed by three washes, they were incubated with HRP-conjugated rabbit anti-porcine IgG for 1 h at 37 °C. Then the wells were washed three times again and handled according to the method of Li *et al.* [43]. Meanwhile, the sera of pigs negative for PRRSV antibodies were used as negative control.

### 4.6. Serum Neutralization Assay

Serum neutralization assays were performed according to previous description [44]. All samples were analyzed in triplicate. The neutralization titers were expressed as log_2_ of the reciprocal of the highest serum dilution in which no cytopathic effects was observed.

### 4.7. Lymphocyte Proliferation Assay

Lymphocyte proliferation assay was performed as previously described [20] with minor modifications. The peripheral blood mononuclear cells (PBMCs) were cultured in RPMI-1640 in 96-well plates at 2 × 10^5^ cells/well in triplicate. Subsequently, the medium was respectively added with 20 μg/mL extracts of PRRSV-infected MARC-145 cells concentrated by ultracentrifugation at 80,000 × *g* for 2 h, with the cell lysate extracted from mock-infected MARC-145 as negative control. Meanwhile, 5 μg/mL ConA was added to a plate, which was used as the positive control. After incubation for 72 h at 37 °C with 5% CO_2_, the proliferation responses were detected through MTT dye assay. At the end of the incubation, the plates were read at 490 nm. The stimulation index (SI) was calculated as the ratio of the average OD value of wells containing antigen-stimulated cells to the average OD value of wells containing cells cultured with medium alone.

### 4.8. Cytokine Assay

The evaluation of cellular immunity was performed through the detection of IFN-γ and interleukin-2 (IL-2). Cytokine levels of IFN-γ and IL-2 were determined through the commercial cytokine ELISA Kits according to the manufacturer’s instruction (pig IFN-γ BMS671, sensitivity: 5 pg/mL, Bender MedSystems GmbH Campus Vienna Bio-center 2 A-1030 Vienna, Austria; porcine IL-2 ELISA Kit QS42565, sensitivity: 1.0 pg/mL, RapidBio Lab).

### 4.9. Statistical Analysis

All data were presented as mean ± S.D. (standard deviation). The differences in the level of humoral and cellular immune responses between different groups were determined by analysis of variance and *t*-test. All data analyses were conducted by SPSS statistics software, Version 14.0. *P*-value < 0.05 was considered statistically significant.

## 5. Conclusion

Because China has a regulation that PRRSV vaccination-challenge experiment must be performed in bio-safety level-3 laboratory, it is impossible for us to evaluate its protection efficiency. However, we succeeded in illustrating that co-expression of M and IL-18 proteins could show significantly stronger effects on the induction of vaccine-induced virus-specific cellular immune responses than expression of M alone.
